# Supplementary material for: High energy acceptor states strongly enhance exciton transfer between metal organic phosphorescent dyes
Source: Nat Commun. 2020 Mar 10;11:1292. doi: 10.1038/s41467-020-15034-0 (PMC7064524; doi:10.1038/s41467-020-15034-0)
Supplement: Supplementary file 1 — Supplementary Information [file 41467_2020_15034_MOESM1_ESM.pdf]

Supplementary Information: High energy acceptor states strongly enhance exciton transfer between metal organic phosphorescent dyes

Xander de Vries[1], Reinder Coehoorn[1,2], and Peter A. Bobbert[1]
[1] *Department of Applied Physics, Eindhoven University of Technology, P.O. Box 513, NL-5600 MB Eindhoven, The Netherlands*
[2] *Institute for Complex Molecular Systems, Eindhoven University of Technology, P.O. Box 513, NL-5600 MB Eindhoven, The Netherlands*
[3] *Center for Computational Energy Research, P.O. Box 6336, NL-5600 HH Eindhoven, The Netherlands*
(Dated: February 3, 2020)

CONTENTS

Supplementray Note 1: Systematic names of the emitter molecules 2

Supplementary Note 2: Calculation of vibronic couplings 2

Supplementary Note 3: Donor and acceptor spectra 3

Supplementary Note 4: Numerical and graphical Förster radii overview 3

Supplementray Note 5: The effect of energetic disorder 5

References 6

SUPPLEMENTRAY NOTE 1: SYSTEMATIC NAMES OF THE EMITTER MOLECULES

(1) fac-Ir(pmp)$_3$: iridium(III)tris-(N-phenyl,N-methyl-pyridoimidazol-2-yl),

(2) mer-Ir(pmp)$_3$: iridium(III)tris-(N-phenyl,N-methyl-pyridoimidazol-2-yl),

(3) FIrpic: iridium(III)bis[(4,6-difluorophenyl)-pyridinato-N,$C^{2'}$]picolinate,

(4) FIr6: iridium(III)bis(4,6-difluorophenylpyridinato)-tetrakis(1-pyrazolyl)borate,

(5) fac-Ir(ppy)$_3$: iridium(III)fac-tris(2-phenylpyridine),

(6) Ir(ppy)$_2$(acac): iridium(III)bis(2-phenylpyridine)-(acetylacetonate),

(7) Ir(BZQ)$_2$(acac): iridium(III)bis(benzo[h]quinolinato-N,C 2')(acetylacetonate),

(8) Ir(BT)$_2$(acac): iridium(III)bis(2-phenyl benzothio-zolato-N,C 2')(acetylacetonate),

(9) Ir(dpo)$_2$(acac): iridium(III)bis(2,4-diphenyl-oxazolato-1,3-N,C 2')(acetylacetonate),

(10) Ir(npy)$_2$(acac): iridium(III)bis[2-(2-naphthyl)-pyridine](acetylacetonate),

(11) Ir(MDQ)$_2$(acac): iridium(III)bis(2-methyldibenzo[f,h]quinoxaline)(acetylacetonate),

(12) Ir(BTP)$_2$(acac): iridium(III)bis(2-(2'-benzothienyl)pyridinato-N,C 3')(acetylacetonate),

(13) Ir(piq)$_3$: iridium(III)tris(1-phenylisoquinoline),

(14) NIr: iridium(III)bis(1-pyrenyl-isoquinolinato-N,C')(acetylacetonate).

SUPPLEMENTARY NOTE 2: CALCULATION OF VIBRONIC COUPLINGS

The normal mode displacement vector in mass-weighted coordinates of the donor molecules, $\mathbf{K}$, is obtained from the Hessian, $\mathbf{H}$, and the energy gradient, $\bar{\mathbf{g}}$, as calculated in the spin-restricted DFT triplet geometry, as follows

$$\mathbf{K} = -\mathbf{H}^{-1}\bar{\mathbf{g}}. \tag{1}$$

For the acceptor molecules, the displacement vector $\mathbf{K}^{(0)}$ of the pure singlet and triplet states (without inclusion of spin-orbit coupling) is obtained using TD-DFT as implemented in the ORCA package, which uses the augmented Hessian approach [2]. This approach is found to be more stable for the acceptor excited states than the quasi-Newton method used for the donor molecules. The effect of spin-orbit coupling in the normal mode components K_{n_i} of the displacement vector is included by

$$K_{n_i} = \sum_m c_i^m K_{n_m}^{(0)}, \tag{2}$$

where c_i^m are the expansion coefficients of excited state i with spin-orbit coupling in the basis of excited states m without spin-orbit coupling.

SUPPLEMENTARY NOTE 3: DONOR AND ACCEPTOR SPECTRA

Figure 1 shows the normalized squared transition dipole moment spectra of the dye molecules included in this work, acting as donor or acceptor.

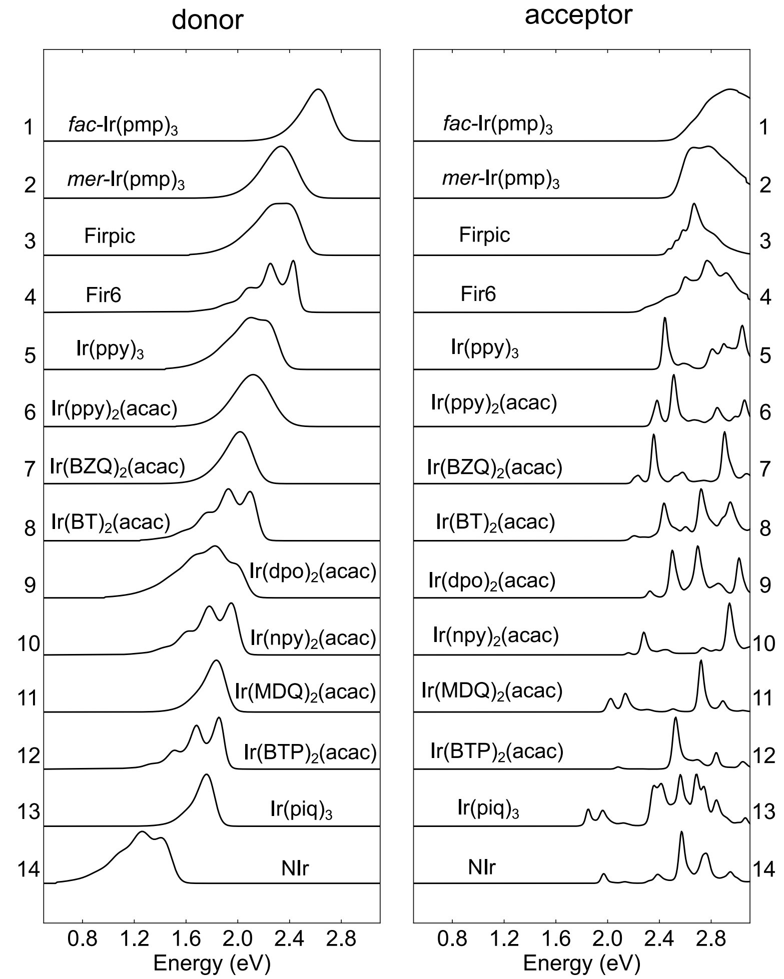

Supplementary Figure 1. Normalized squared transition dipole moment spectra of the dyes, acting as donor (left) and acceptor (right).

SUPPLEMENTARY NOTE 4: NUMERICAL AND GRAPHICAL FÖRSTER RADII OVERVIEW

Table 1 contains the calculated Förster radii for donor-acceptor transfer, for all 84 combinations of dyes for which this transfer is possible. The results are graphically shown in Fig. 4 in the main text. Figure 2 shows the Förster radii of the 84 combinations, plotted separately per dye that can act as donor.

Supplementary Table 1. Förster radii for transfer between dyes, acting as donor (rows) and acceptor (columns). No energetic disorder is assumed and $\kappa_{ij}^2 = \langle\kappa\rangle^2 = 0.67$. The values are displayed graphically in Fig. 4 in the main text.

R_{F}[nm]	*fac*-Ir(pmp)$_3$	*mer*-Ir(pmp)$_3$	Firpic	Fir6	Ir(ppy)$_3$	Ir(ppy)$_2$acac	Ir(BZQ)$_2$acac	Ir(BT)$_2$acac	Ir(dpo)$_2$acac	Ir(npy)$_2$acac	Ir(MDQ)$_2$acac	Ir(BTP)$_2$acac	Ir(piq)$_3$	NIr
fac-Ir(pmp)$_3$	2.139	2.185	2.327	1.785	2.335	2.406	2.257	2.604	2.942	2.122	2.536	2.716	3.238	3.824
mer-Ir(pmp)$_3$	-	1.334	1.540	1.470	2.308	2.305	2.513	2.396	2.402	2.414	2.363	2.167	3.041	3.096
Firpic	-	-	1.397	1.409	2.238	2.209	2.449	2.333	2.271	2.367	2.467	2.010	2.978	3.002
Fir6	-	-	-	1.467	2.448	2.264	2.393	2.448	2.463	2.426	2.426	2.132	3.009	2.989
Ir(ppy)$_3$	-	-	-	-	1.271	1.582	2.120	1.904	1.671	2.228	2.666	1.644	2.654	2.797
Ir(ppy)$_2$acac	-	-	-	-	-	1.606	2.057	1.883	1.653	2.114	2.651	1.667	2.611	2.774
Ir(BZQ)$_2$acac	-	-	-	-	-	-	1.376	1.399	0.804	1.473	2.729	1.689	2.799	3.028
Ir(BT)$_2$acac	-	-	-	-	-	-	-	1.361	0.672	1.426	2.627	1.653	2.733	2.881

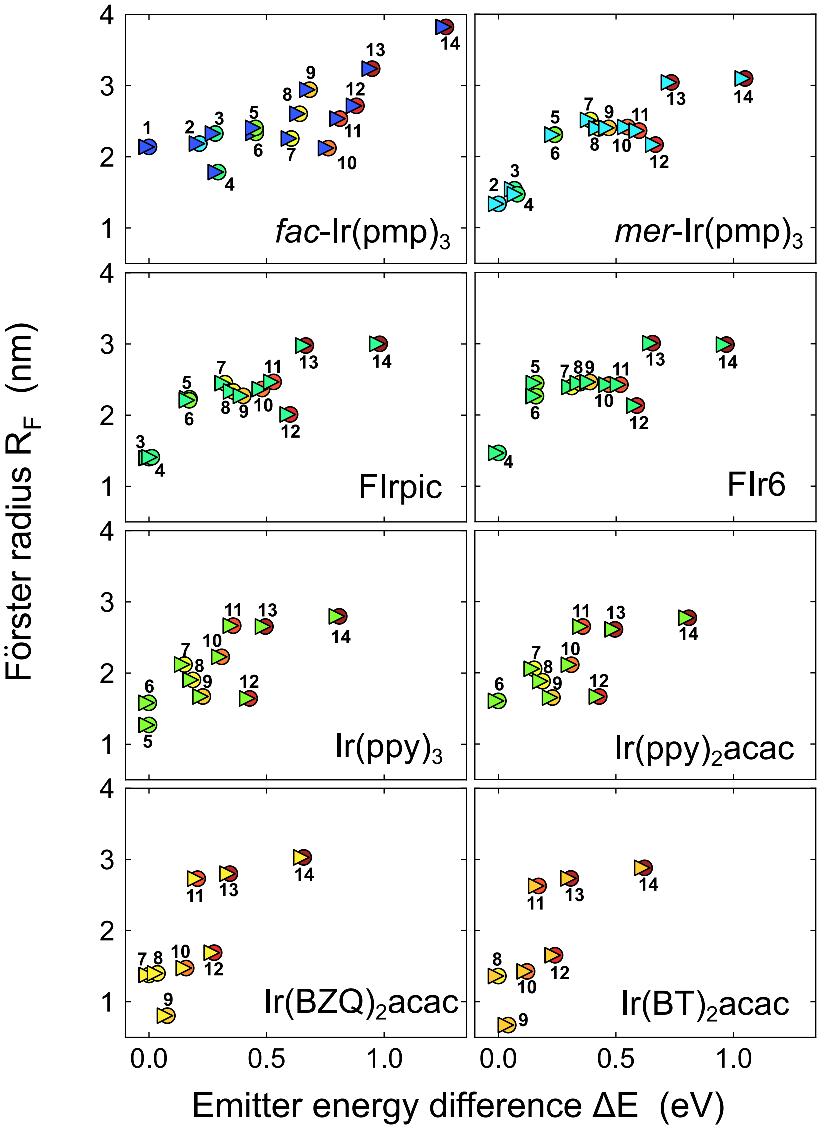

Supplementary Figure 2. Overview of the calculated Förster radius versus donor-acceptor energy difference (exothermicity) for the 84 dye combinations, plotted separately for the different dyes that can act as donor. Each point in the graph represents a transfer process from a dye acting as donor (triangle) to a dye acting as acceptor (circle), with emission colors as indicated.

SUPPLEMENTRAY NOTE 5: THE EFFECT OF ENERGETIC DISORDER

Figure 3 shows how the Förster radii are influenced by the energetic disorder. Here, the Förster radii were calculated from the averaged transfer rate for a set of 100 donor-acceptor combinations with energies of donor and acceptor drawn from a Gaussian distribution with disorder parameter $\sigma = 0$ eV (redrawn from the main paper), $\sigma = 0.05$ eV and $\sigma = 0.10$ eV. With increasing disorder the scatter in the Förster radii tends to decrease. For isoenergetic transfer ($\Delta E \approx 0$) the Förster radius is enhanced. This can be explained by the fact that the Gaussian distribution broadens the spectra and increases spectral overlaps, leading to larger Förster radii. In realistic systems the energetic disorder is expected to be around 0.05 eV [1]. We see that the effect of the disorder is then weak for a single transfer step.

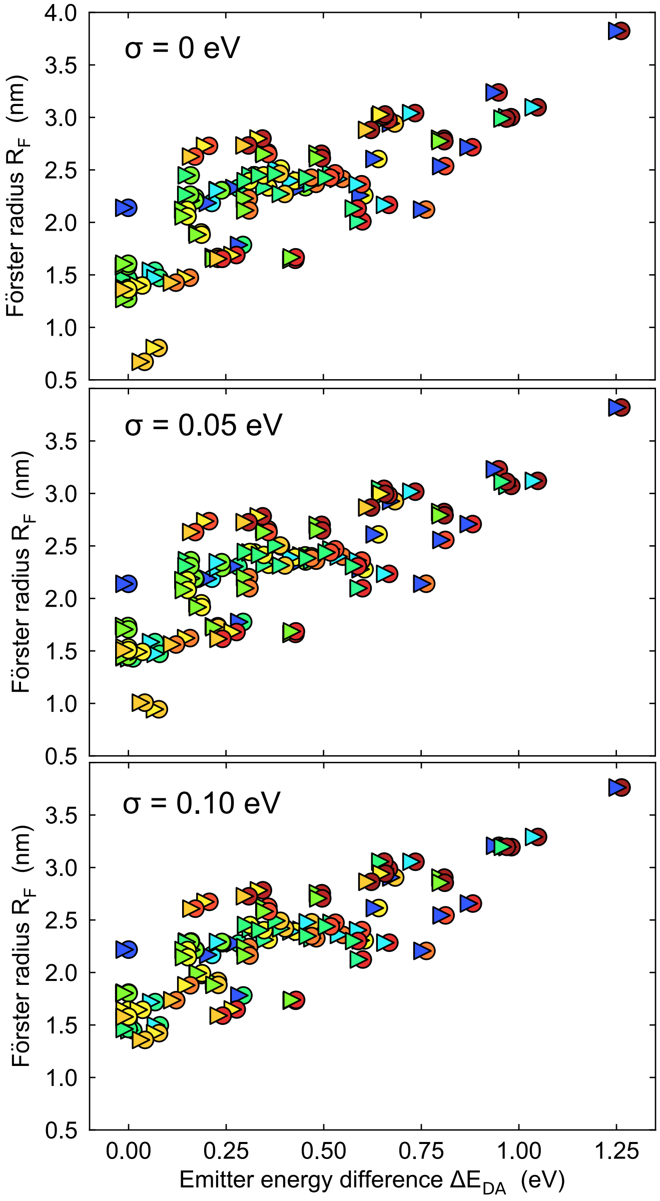

Supplementary Figure 3. Förster radii versus donor-acceptor energy difference, for three values of the width (standard deviation) of a Gaussian distribution of triplet state energies, $\sigma = 0$ eV (redrawn from the main paper), $\sigma = 0.05$ eV and $\sigma = 0.10$ eV. Each point in the graph represents a donor-acceptor pair, where the triangle has the donor emission color and the circle the acceptor emission color.

[1] Vries, X. D., Friederich, P., Coehoorn, R. & Bobbert, P. A. Triplet exciton diffusion in metal-organic phosphorescent host-guest systems from first principles. *Phys. Rev. B* **99**, 205201 (2019).

[2] De Souza, B., Neese, F. & Izsák, R. On the theoretical prediction of fluorescence rates from first principles using the path integral approach. *J. Chem. Phys.* **148** (2018).
